# M Protein of Group a Streptococcus Plays an Essential Role in Inducing High Expression of A20 in Macrophages Resulting in the Downregulation of Inflammatory Response in Lung Tissue

**DOI:** 10.3389/fcimb.2018.00131

**Published:** 2018-05-11

**Authors:** Cuiqing Ma, Xue Gao, Shuhui Wu, Ling Zhang, Jiachao Wang, Zhengzheng Zhang, Zhiyan Yao, Xiaotian Song, Wenjian Li, Xiurong Wang, Huidong Feng, Lin Wei

**Affiliations:** ^1^Key Laboratory of Immune Mechanism and Intervention on Serious Disease in Hebei Province, Department of Immunology, Basic Medical College, Hebei Medical University, Shijiazhuang, China; ^2^Department of Microbiology and Immunology, Hebei University of Chinese Medicine, Shijiazhuang, China

**Keywords:** Group A Streptococcus (GAS), macrophages, A20, M protein, negative regulation

## Abstract

Group A streptococcus (GAS), a common pathogen, is able to escape host immune attack and thus survive for longer periods of time. One of the mechanisms used by GAS is the upregulated expression of immunosuppressive molecules, which leads to a reduction in the production of inflammatory cytokines in immune cells. In the present study, we found that macrophages produced lower levels of proinflammatory cytokines (IL-1β, TNF-α, IL-6) when challenged with GAS than they did when challenged with *Escherichia coli* (*E. coli*). Simultaneously, in a mouse model of lung infection, GAS appeared to induce a weaker inflammatory response compared to *E. coli*. Our data also indicated that the expression of the A20 transcriptional regulator was higher in GAS-infected macrophages than that in macrophages infected with *E. coli*, and that high expression of A20 correlated with a reduction in the production of TRAF6. SiRNA targeting of A20 led to the increased production of TRAF6, IL-1β, TNF-α, and IL-6, suggesting that A20 inhibits synthesis of these key proinflammatory cytokines. We also investigated the pathway underlying A20 production and found that the synthesis of A20 depends on My88, and to a lower extent on TNFR1. Finally, we showed a significant reduction in the expression of A20 in macrophages stimulated by M protein-mutant GAS, however, a speB-GAS mutant, which is unable to degrade M protein, induced a greater level of A20 production than wild type GAS. Collectively, our data suggested that M protein of GAS was responsible for inducing A20 expression in macrophages, which in turn down-regulates the inflammatory cytokine response in order to facilitate GAS in evading immune surveillance and thus prolong survival in the host.

## Introduction

Group A streptococcus (GAS), also referred to as *Streptococcus pyogenes*, is the most common and versatile of human pathogens. Furthermore, GAS is responsible for a wide spectrum of human diseases, ranging from superficial skin infections to lethal diseases (Cunningham, 2000; Bryant et al., 2003; Liang et al., 2012). In particular, GAS is known to survive for long periods in hosts and for recurrent attacks, particularly in the elderly and children. However, the strategy of how GAS is able to stay alive in hosts for long periods is not clear. Previous studies have reported that GAS exploits complement regular factors to bind surface proteins, such as M and FbaA protein (Terao et al., 2001; Pandiripally et al., 2003; Ma et al., 2011), or to promote the cleavage of cell surface-bound C3b to iC3b to inhibit opsonization by C3, which in turn promotes bacterial entry into epithelial cells and protects against antibiotics (Cue et al., 1998; Collin and Olsén, 2002; Collin et al., 2002). As the first line of defense in innate immunity, macrophages can trigger an inflammatory response following the recruitment of neutrophils and more macrophages, to eradicate invasive pathogens. However, the mechanisms underlying the way in which GAS can escape innate immunity cell attack and survive in the body are poorly defined. We speculated that GAS should have a reverse effect on macrophages and be able to inhibit their function. Our previous experiments have screened a range of negative regulatory proteins such as SOCS1, SOCS2, COCS3, and A20 in macrophages following GAS infection and found that both SOCS1 (Wu et al., 2015) and A20 were activated. However, the mechanism responsible for the inhibition of A20 is different from that of SOCS1. In the present study, we focused on the interaction between GAS and A20 in macrophages.

A20 is an important negative regulator of immune cells, and its expression can be induced by a wide variety of stimuli, including tumor necrosis factor (TNF), interleukin (IL)-1, phorbol esters, hydrogen peroxide, lipopolysaccharide (LPS), viruses, and the cell surface receptor CD40. A20 can alsorestrain nuclear factor-κB signals mediated by Toll-like receptors, tumor necrosis factor receptor 1 (TNFR1), and NOD2 (nucleotide oligomerization domain-2)-like receptor to create feedback that inhibits the excessive release of TNF-α, IL-1, IL-6, IL-8, intercellular adhesion molecules, and other proinflammatory factors. In this manner, A20 can control cell apoptosis and necrosis (Opipari et al., 1992; Wertz et al., 2004; Hitotsumatsu et al., 2008).

In this study, we found GAS induced lower levels of p65, a key transcription factor of inflammatory cytokines, and inflammatory factors in macrophages, when compared to those inducedby *E. coli*. In addition, A20 expression induced by GAS appeared earlier and was higher than that induced by *E. coli*. GAS is known to produce a variety of exocrine proteins and express a series of cell surface proteins. Therefore, we attempted to identify the probable molecular mechanisms of interaction between GAS and A20, and in particular, which major components of GAS were involved in the production of A20 by macrophages.

## Materials and methods

### Mice

Female C57BL/6 mice, aged 7–8 weeks, were purchased from Beijing Laboratory Animal Center. MyD88^−/−^ mice were also purchased from the Model Animal Research Center of Nanjing University. All mice were housed and manipulated in accordance with the Care and Use of Laboratory Animals (China), and were maintained under specific pathogen-free conditions. The protocol for animal experiments was approved by the animal experimental ethics committee of Hebei Medical University (Reference number: 2015046).

### Bacteria

Streptococcal strain GAS SF370 M1 and other mutant GAS were stored in our laboratory at −80°Cand routinely grown at 37°C in Todd-Hewitt Broth supplemented with 0.5% yeast extract (THY) (Difco, MI, USA). In brief, cryopreserved GAS were reanimated, inoculated into agar plates containing sheep blood, and incubated at 37°C for 18 h. A single colony was then transferred to 3 ml of THY broth at 37°C and shaken overnight at 250 rpm. Finally, the bacteria were collected in 1.5-ml microcentrifuge tubes by centrifugation at 4,000 rpm for 5 min, washed three times, resuspended in 1 ml phosphate-buffered saline (PBS), and counted. *E. coli* was routinely cultivated at 37°C in LB culture media.

### Cells

The murine macrophage cell line RAW264.7 (RAW cells) was purchased from the Cell Centre of Chinese Academy of Medical Sciences and was maintained in DMEM supplemented with 100 U/ml penicillin and streptomycin, and 10% FBS (Gibco BRL, USA) (complete medium). Mouse bone marrow-derived macrophages (BMDMs) were generated from bone marrow cells obtained from the femurs of 15-week-old wild type (WT) or MyD88^−/−^ C57 BL/6 mice. Bone marrow cells (3 × 10^6^) were cultured with DMEM complete medium containing 10 ng/ml M-CSF. After culturing for 7 days, the fully differentiated BMDMs were used for experiments.

### GAS infection of macrophages

RAW cells or BMDMs were seeded at 1 × 10^6^/well in 6-well plates containing culture medium without antibiotics. The next day, GAS and *E. coli* were harvested during the mid-logarithmic growth phase and added to cells at a multiplicity of infection (MOI) equal to 10. After 2 h of incubation at 37°C, non-adherent extracellular bacteria were eliminated by removing the culture medium and washing using phosphate buffer saline (PBS). Adherent extracellular bacteria were subsequently killed by incubation with fresh medium containing 10 μg/mL penicillin G/streptomycin at 37°C for 2 h. At specific time points after infection, supernatants were collected for ELISA, and the cells were prepared for quantitative real-time polymerase chain reaction (qPCR) or Western blot analysis.

### qPCR

Cells collected at various time points were used to isolate total RNA using the RNeasy kit (Takara, Bio. Inc., Japan) in accordance with the manufacturer's protocol. qPCR analysis was then performed using oligo (dT) and random primers by a modified protocol. In brief, cDNA samples converted from 1 μg total RNA was diluted at several concentrations. Diluted cDNA was mixed with a pair of primers (10 μM) targeting mouse IL-1β, IL-6, TNF-α, and β-actin cDNA sequences at an annealing temperature of 60°C and with 35 amplification cycles following the manufacturer's instructions. The following primers were used for PCR amplification: IL-6-F: AAG GAG TGG CTA AGG ACC AA; IL-6-R: GTT TGC CGA GTA GAT CTC AAA; IL-1β-F: TTC CTT GTG CAA GTG TCT GAA G; IL-1β-R: CAC TGT CAA AAG GTG GCA TTT; TNF-α-F: TGA CGT GGA ACT GGC AGA AGA; TNF-α-R: TGG GCC ATA GAA CTG ATG AGA G; β-actin-F: TAC CCA GGC ATT GCT GAC AGG; β-actin-R: ACT TGC GGT GCA CGA TGG A. The 2-ΔΔCt method of relative quantification was used to calculate changes in the expression of target genes (Kubista et al., 2006). Data are presented as the mean ± standard deviation of triplicate samples and are representative of three independent experiments.

### ELISA

Supernatants from RAW cells were harvested at different points after GAS stimulation and stored at −70°C, and then enzyme-linked immunosorbent assay (ELISA, eBioscience Inc. USA) was performed to measure the expression of IL-6, TNF-α, or IL-1β, in accordance with the manufacturer's instructions. In brief, 50 μl of each sample, and 50 μl of sample diluents, were added into pre-washed wells and a standard dilution was added to all standard wells. Subsequently, 50 μl biotin-conjugate was added to all wells followed by incubation at room temperature for 2 h. After washing, streptavidin-HRP and then TMB substrate solution were successively added to each well. The absorbance of each well was then read at a wave length of 450 nm. Data are presented as the mean ± standard deviation of triplicate samples and are representative of three independent experiments.

### Histology

C57BL/6 mice were anesthetized with 1% pentobarbital sodium, and challenged by intranasal (i.n.) instillation of 10^8^ CFU 50% tissue culture infective doses (TCID50)/50 μl of GAS or *E. coli*, with PBS acting as a negative control. After 48 h, the treated mice were sacrificed and the right lung was isolated and immediately fixed in 4% paraformaldehyde. The left lung was perfused with 1 ml PBS to collect bronchoalveolar lavage fluid (BALF), which was centrifuged. Most of the supernatant was removed and cell smears of centrifugal sedimentation were stained with Wright Giemsa. The number of leukocytes present was then counted under oil microscopy. Tissue samples were subsequently processed, embedded in paraffin, thin sectioned, and placed on slides coated with L-lysine. Sections were stained with hematoxylin and eosin (H&E) and periodic acid Schiff (PAS) to demonstrate the inflammatory response.

### Western blotting

Total cell protein was extracted from cells infected by GAS or *E. coli* using the RIPA protein extraction kit (Beyotime, CHN) and was then used for Western blotting to calculate the changes in A20, TRAF6, and p-P65. In brief, cells infected by GAS or *E. coli* were washed twice, mixed with RIPA lysis buffer, and then centrifuged at 8,000 g for 5 min at 4°C. Then, the supernatant was collected and protein concentration was determined according to the Bradford method (Bradford, 1976). Total cell lysates or M protein were then separated using sodium dodecyl sulfate polyacrylamide gel electropheresis (SDS-PAGE) and transferred to polyvinylidene difluoride (PVDF) membrane. After blocking, blots were incubated with rabbit monoclonal antibodies against A20/TNFAIP3 (Cell signaling Inc.), TRAF6 (Cell signaling Inc. USA), and p-NF-κB p65 (Cell signaling Inc. USA); β-actin (Cell signaling Inc.) was used as an internal control. Finally, blots were hybridized with horseradish-peroxidase-(HRP-)-conjugated goat anti-rabbit immunoglobulin G (IgG), incubated with enhanced chemiluminescence (ECL) solution (PerkinElmer Life Sciences, USA), and finally exposed to X-ray film.

### SiRNA

For SiRNA treatment, 2 × 10^5^ RAW264.7 cells were seeded per well on a six-well tissue culture plate for 18 h at 37°C in a CO_2_ incubator. SiA20 transfection medium was then added to the pre-washed cells in accordance with a siRNA transfection protocol (Santa Cruz Biotechnology, Inc. USA) and then incubated for 6 h at 37°C in a CO_2_ incubator. After the transfection mixture was removed, the cells were stimulated with heat-killed GAS for 7 h, and live GAS for 1 h, respectively. Finally, the supernatant of the transfected cells was collected for ELISA. Cells were washed once with PBS, and then fixed with 4% paraformaldehyde for immunoflurescence studies, or harvested for Western blot analysis or qRT-PCR analysis.

### Immunoflurescence

Cells were grown on glass cover slips and incubated overnight at 37°C and 5% CO_2_ in an incubator. After being washed four times, cells were fixed in 4% paraformaldehyde and blocked in 1% BSA (Saint Louis, USA) for 1 h at 37°C. Subsequently, cells were incubated with monoclonal anti-p-P65 (1: 100) and anti-A20 (1:100) antibodies overnight at 4°C. After four rinses, FITC-conjugated and secondary antibodies were used to visualize the target proteins by fluorescence microscopy (Tokyo, Japan). Nuclei were counter-stained with DAPI.

### Statistical analysis

SPSS statistical software (version 21.0) was used for data analysis. The standard deviation (SD) of the mean is shown unless otherwise indicated. Statistical significance is indicated as either ^*^p < 0.05 or ^**^p < 0.01.

## Results

### Gas induced a weaker inflammatory reaction than *E. coli*

**Figure 1:**
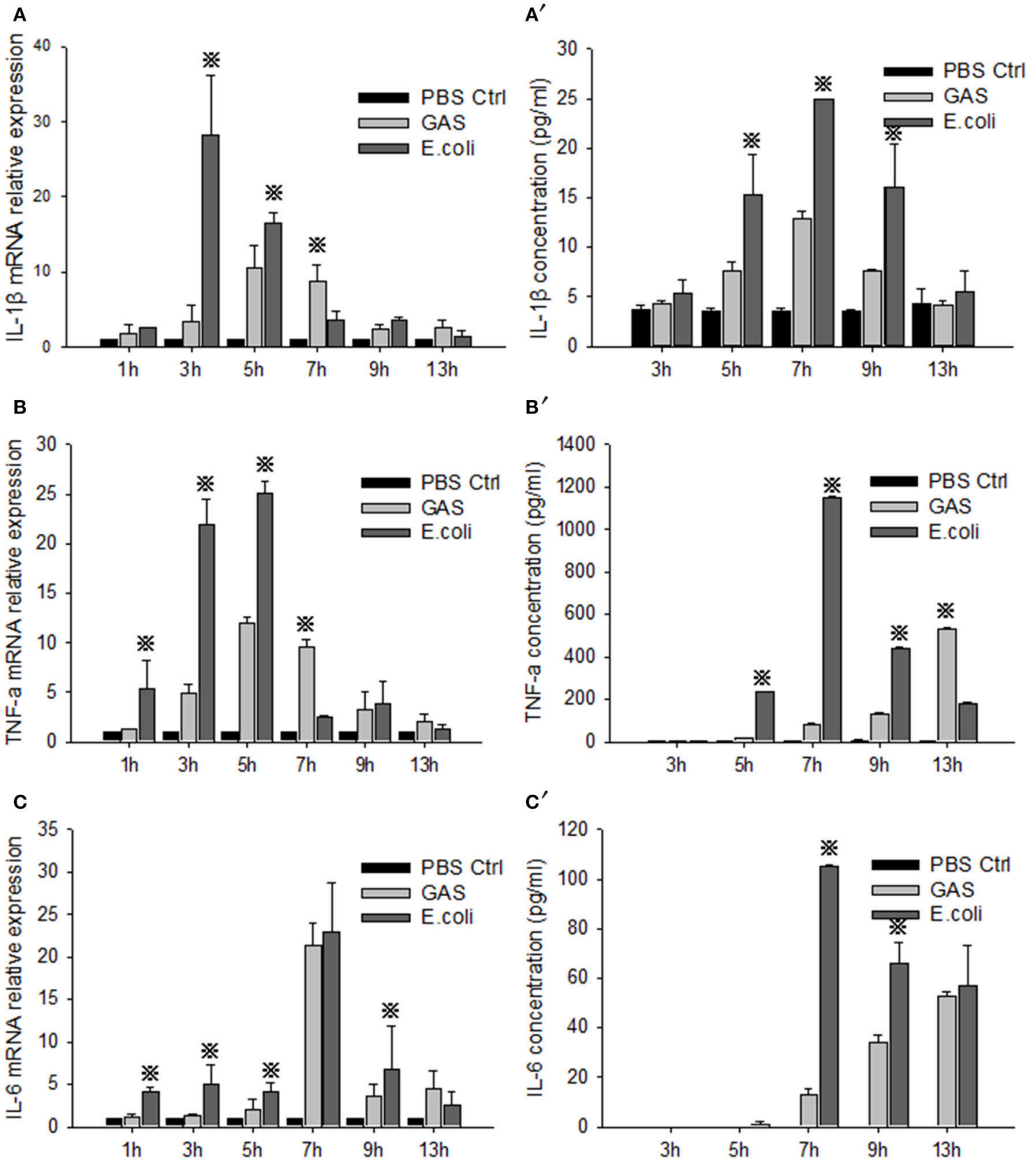
Evaluation of proinflammatory cytokines in RAW cells stimulated with GAS or *E. coli*. RAW264.7 cells were seeded at a concentration of 1 × 10^6^/well in a 6-well plate containing medium without antibiotics overnight and then infected with GAS, or *E. coli* as a control, at an MOI of 10. We then collected supernatant and cells from each group at different time points. Total RNA was isolated from cells using RNeasy, and qPCR analysis was performed to detect the mRNA level of IL-1β **(A)**, TNF-α **(B)**, and IL-6 **(C)**. Simultaneously, ELISA was performed to detect the protein expression of IL-1β **(A**′**)**, TNF-α **(B**′**)**, and IL-6 **(C**′**)** in the cell supernatant. Each experiment was performed at least three times. Results are presented as mean ± SD. *P* < 0.05 vs. *E. coli* group.

RAW cells were stimulated by GAS or *E. coli*, respectively, and the proinflammatory cytokines IL-1β, TNF-α, and IL-6, were detected by qRT-PCR and ELISA at different time points. The relative mRNA expression levels of IL-1β, TNF-α, and IL-6 in GAS-infected RAW cells were all weaker than those in *E. coli*-infected RAW cells at 1, 3, and 5 h post-stimulation (*P* < 0.05) (Figures 1A–C). Accordingly, the protein levels of these proinflammatory cytokines were also lower than those produced by *E. coli*-infected RAW cells until 9 h post-stimulation (*P* < 0.05) (Figures 1A′–C′), suggesting that GAS induced a lower level of proinflammatory cytokine reaction in RAW cells than *E. coli* at an early stage after infection.

**Figure 2:**
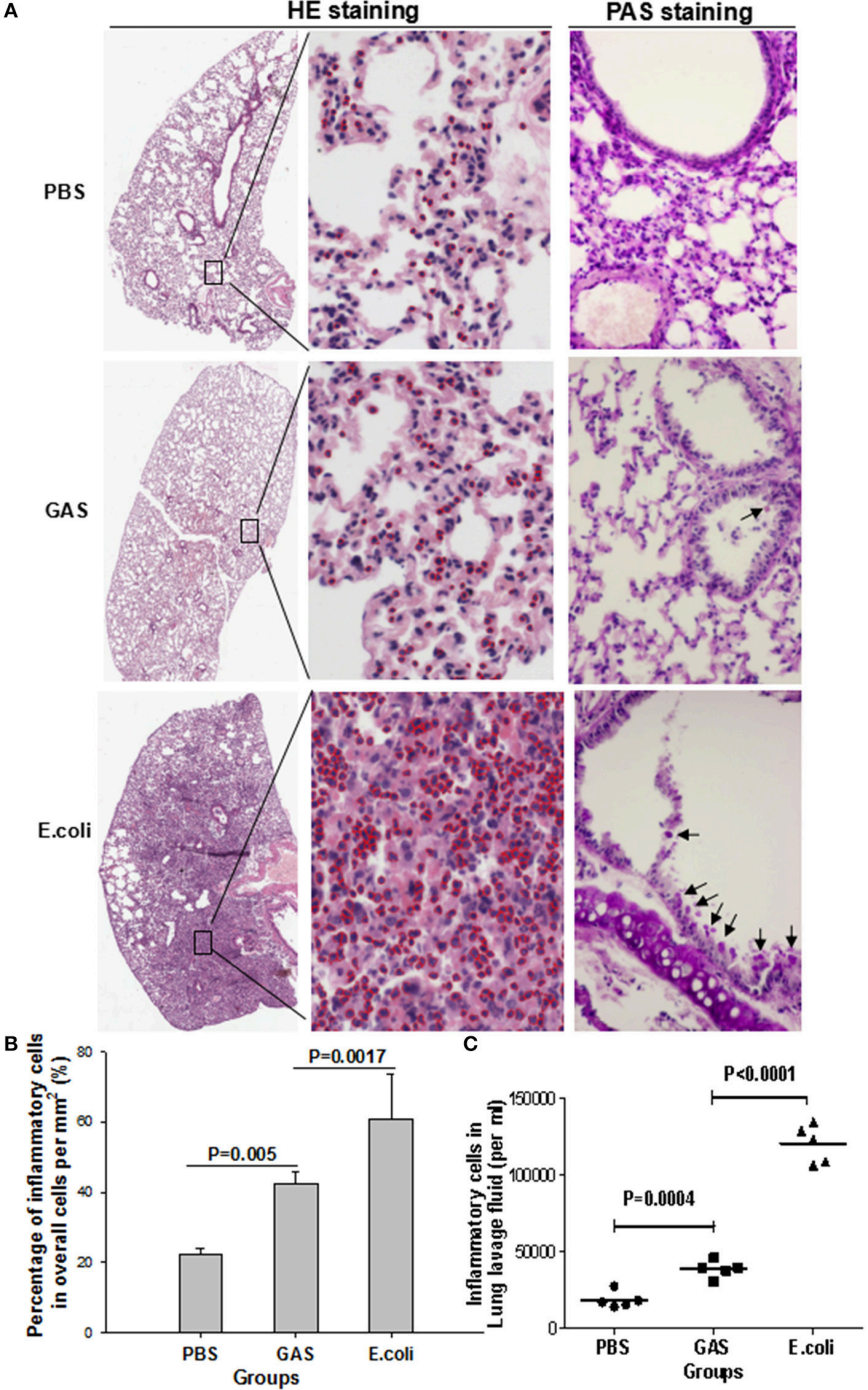
Histological evaluation of inflammatory response in mouse lung tissue following GAS or *E. coli* infection. The three experimental groups of mice were separately prepared for histopathological analysis. **(A)** Representative views of lung sections treated with PBS, GAS, or *E. coli*. In the left panels, H&E staining shows the overall outline of lung slices, with a magnified image. Inflammatory cells are identified by red circles in the middle panels (H&E staining, × 10 and ×40). Periodic acid–Schiff (PAS) staining showed bronchiolar mucus production in each group (right panels, objective ×20). **(B)** Quantitative analysis of recruited inflammatory cells per square mm. **(C)** Inflammatory cells were counted in lung lavage fluid (per ml). Each experiment was repeated three times, and similar results were obtained on each occasion.

It was not clear whether these lower levels of proinflammatory cytokines would induce a weaker state of inflammation in response to GAS compared with *E. coli*. We therefore tested this in a mouse model infected with 1 × 10^7^ CFU of GAS or *E. coli*, or with PBS as a control for 48 h, and then performed histopathological analysis using H&E staining and PAS staining. As expected, H&E staining showed that the *E. coli*-challenged lungs of mice contained abundant inflammatory cell infiltration in the alveolar walls, while PAS staining revealed intra-alveolar edema, and hemorrhage, accompanied by bronchiolitis with shed epithelium and mucus in the bronchus (Figures 2A,B). In contrast, GAS-challenged lungs showed a dramatic reduction of inflammatory cells along with reduced levels of mucus and alveolitis (Figures 2A,B). Lungs from the group of mice treated with PBS showed infiltration of only a few inflammatory cells and no mucus (Figures 2A,B). There were far fewer inflammatory cells in the BALF of the GAS group compared to the *E. coli* group (*P* < 0.0001), although, there was a significant difference between the GAS group and the PBS control (*P* = 0.0004; Figure 2C). These results indicated that when challenged with GAS, mice elicited a weaker inflammatory response compared with mice challenged with *E. coli*.

### A20 expression was stronger and lasted longer in GAS-stimulated RAW cells and BMDM than that of *E. coli*

**Figure 3:**
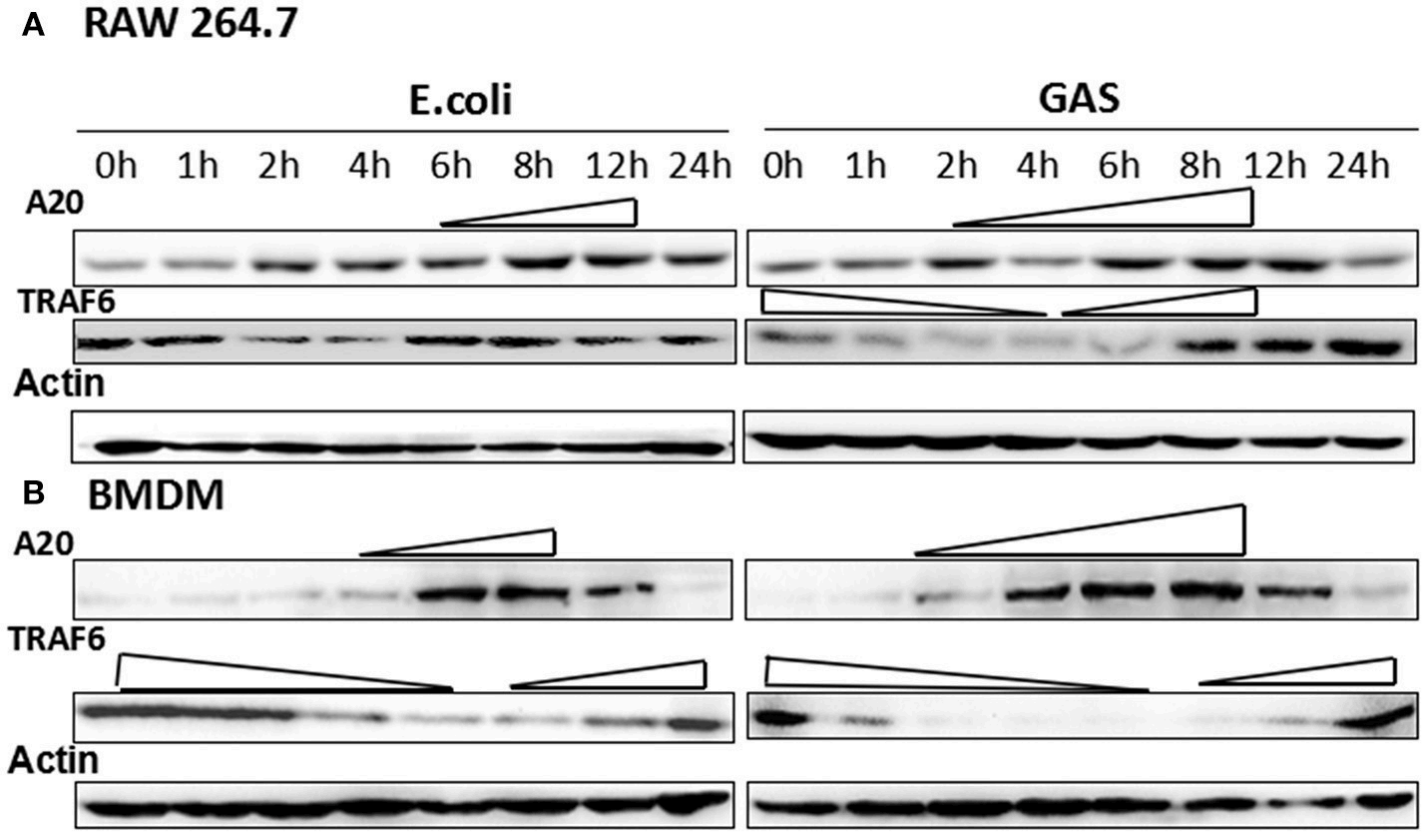
Expression of A20 and its target protein, TRAF6, in GAS-induced macrophages. Macrophages from wild-type mice were infected with GAS or *E. coli* at an MOI of 10. At different post-infection times, the protein levels of A20 and TRAF6 were detected by Western blot in RAW 264.7 cells **(A)** or BMDMs **(B)**. Each experiment was repeated three times, and similar results were obtained on each occasion.

RAW cells were harvested following infection with GAS or *E. coli* (MOI of 10:1) at different time points, and the expression of A20 was analyzed by Western blotting. The results showed that A20 expression gradually increased in RAW cells from 2 h and peaked at 6–8 h following stimulation by GAS (Figure 3A). Accordingly, the expression of TRAF6, the down-stream target protein of A20, was reduced from 1 to 6 h and increased again from 8 h. The expression of A20 in RAW cells stimulated by *E. coli* was delayed and was lower than that of cells stimulated by GAS. Accordingly, TRAF6 expression fell from 2 to 4 h and then increased gradually from 6 h. We also investigated these mechanisms in BMDM cells (Figure 3B) and observed a similar pattern of results. Consequently, the expression of A20 expression in GAS-infected macrophages was higher and lasted longer than that of BMDM cells infected with *E. coli*; furthermore, the levels of TRAF6 were lower than those of BMDW cells infected with *E. coli*.

**Figure 4:**
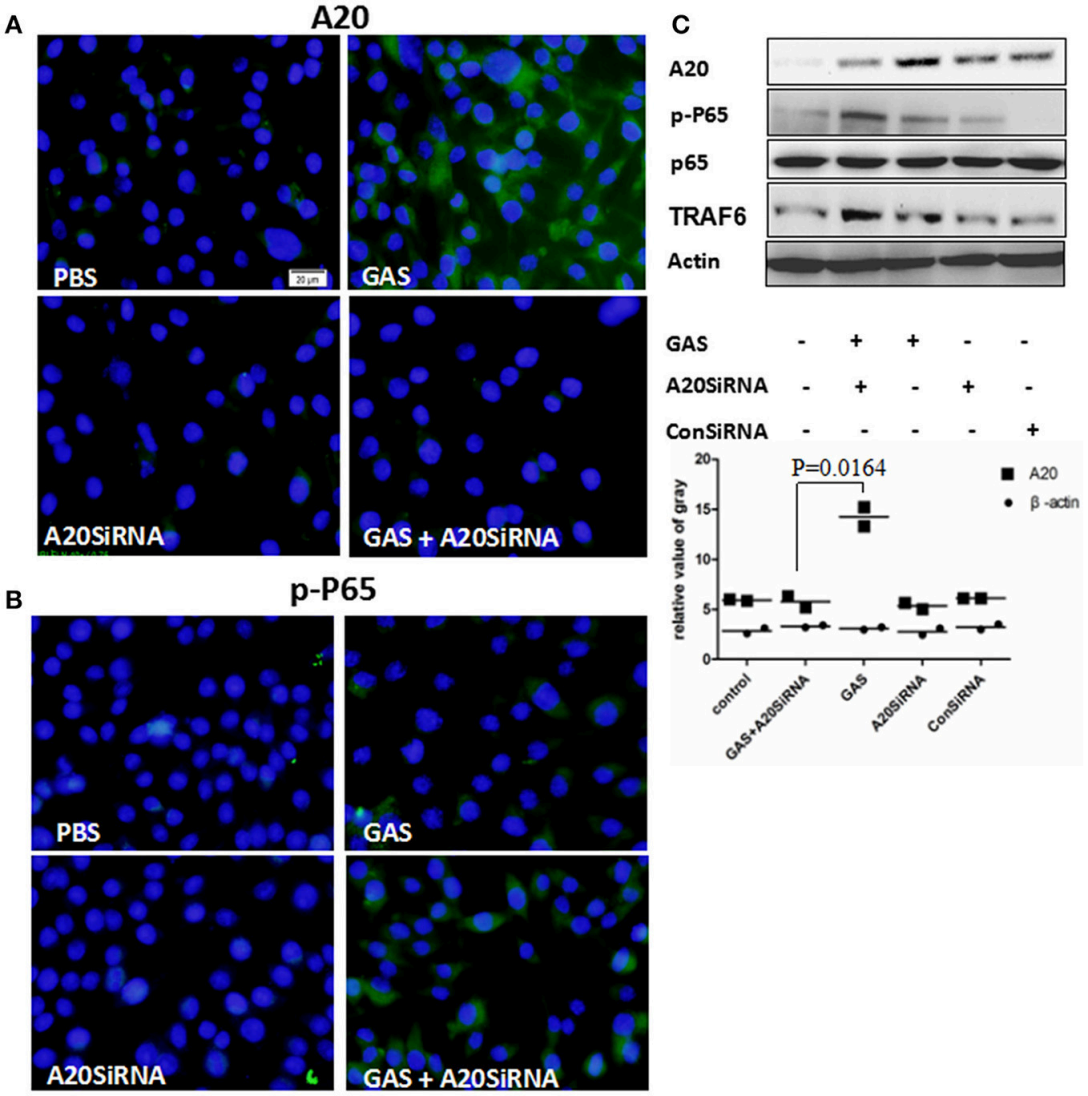
Expression of A20 and the phosphorylation of p65 in RAW 264.7 macrophages with and without A20 SiRNA following GAS infection. RAW 264.7 cells were incubated with A20 SiRNA for 30 min, and then infected with GAS at an MOI of 10: 1. Six hours post-infection, an immunofluorescence assay (magnification, 3100) was performed to detect the expression of A20 (Green) **(A)** and p-P65 (Green) **(B)**, which was verified by Western blotting **(C)**. qPCR (for mRNA expression) or ELISA (for protein expression) were also used to detect the levels of proinflammatory cytokines in RAW cells or in the supernatant (Figure 5). The blue color represents DAPI staining. Representative cells from the same field for each group are shown. Each experiment was repeated three times, and similar results were obtained on each occasion.

**Figure 5:**
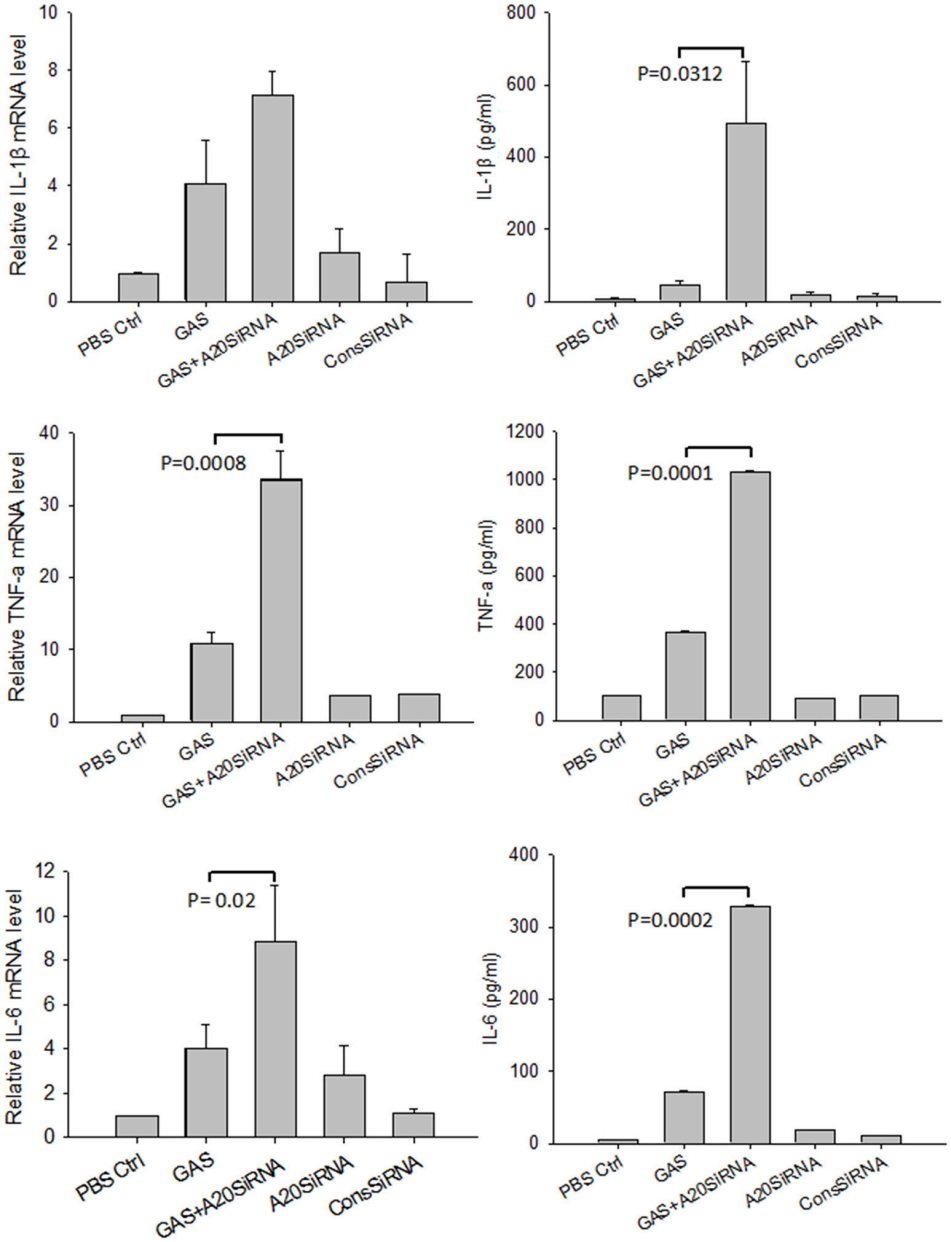
Expression of proinflammatory cytokines in RAW 264.7 macrophages with and without A20 SiRNA following GAS infection. RAW 264.7 cells were incubated with A20 SiRNA for 30 min, and then infected with GAS at an MOI of 10. And 6 h post-infection, ELISA and RT-PCR were performed to analyze the expression of proinflammatory cytokines. Each experiment was repeated three times, and similar results were obtained on each occasion. Results are presented as mean ± SD.

Next, we attempted to use an A20 SiRNA assay to confirm the above results. Following silencing of the A20 gene by SiA20 (Figures 4A,C), the level of TRAR6 clearly increased (Figure 4C). Correspondingly, p-P65 activation increased significantly (Figures 4B,C) in GAS-stimulated RAW cells for 6 h. Simultaneously, the expression levels of IL-1β, TNF-α, and IL-6 in the supernatant of GAS-stimulated RAW cells were all significantly enhanced compared with other groups (Figure 5). These results suggested that A20, a negative regulatory protein, is likely to play an essential role in inhibiting the expression of inflammatory factors in macrophages following GAS infection.

### GAS-induced A20 expression in macrophages mainly depended on the MyD88-signaling pathway but partially depended on TNFR1

**Figure 6:**
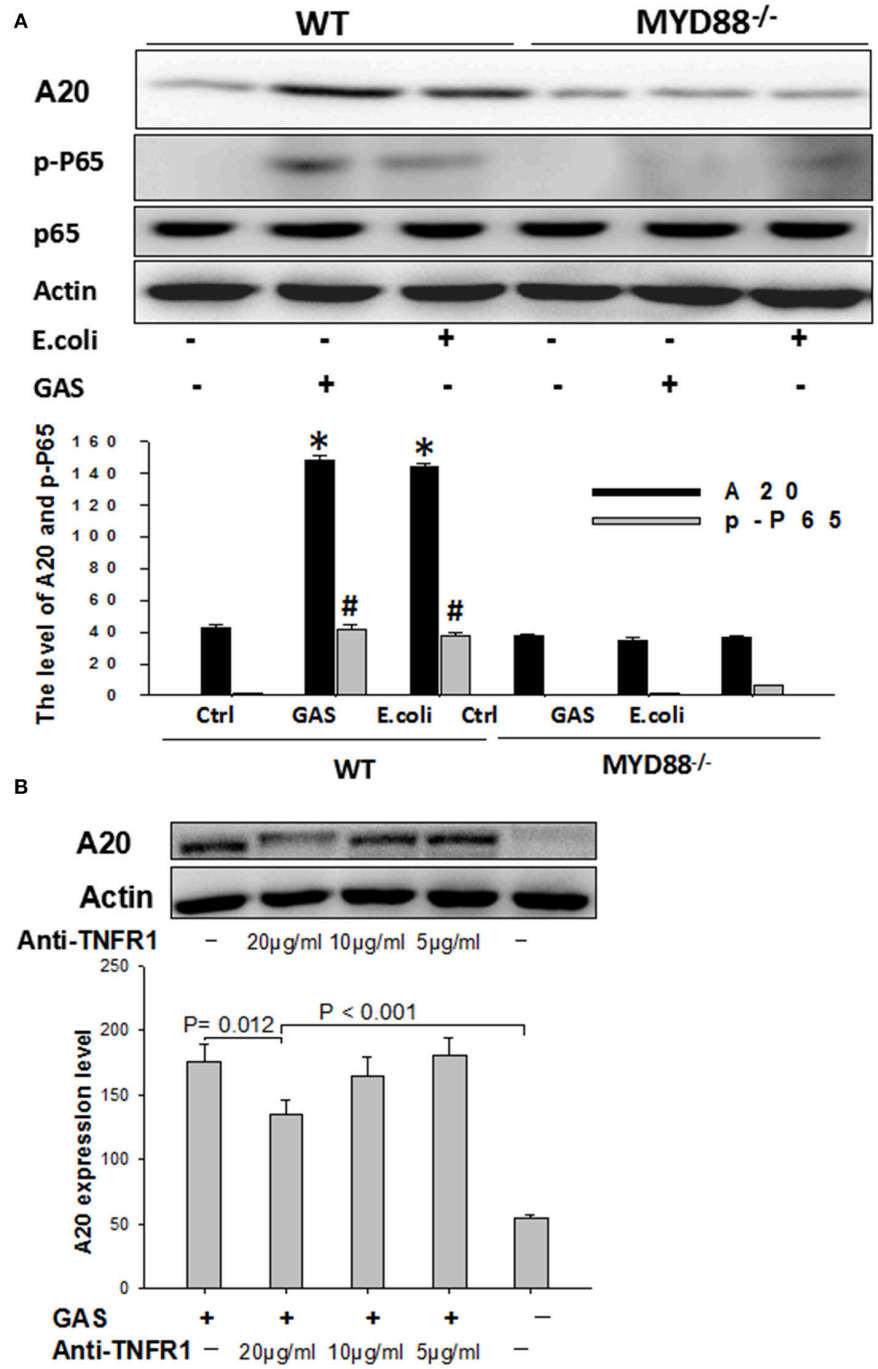
Western blot analysis of A20 expression in wild type or MyD88-/- BMDMs stimulated with GAS, and the detection of A20 expression following blockade with anti-TNFR1. BMDMs from MyD88^−/−^ knockout mice were stimulated with GAS at an MOI of 10. Six hours later, the treated cells were collected and determined by Western blotting using antibodies targeted to A20, p-P-65, and β-actin **(A)**. ^*^P < 0.05 and ^#^ *P* < 0.001 vs. MyD88^−/−^ group. RAW cells were blocked with anti-TNFR1 monoclonal antibody for 30 min and then stimulated with GAS at an MOI of 10 for 6 h, following collection of the treated cells, A20 expression was detected by Western blotting **(B)**. Each experiment was repeated three times, and similar results were obtained on each occasion.

In order to determine whether MyD88 was involved in the expression of A20, BMDM cells, harvested from MyD88^−/−^knock-out mice, were infected with either GAS or *E. coli* for 6 h and then total cell protein was analyzed by Western blotting using WT BMDM cells as a control. As expected, the expression of A20 was much lower in MyD88^−/−^ BMDM cells than in WT groups (*P* < 0.05); furthermore, we failed to detect the downstream protein p-P65, in MyD88^−/−^ BMDM cells stimulated by GAS (Figure 6A), suggesting that the expression of A20 is mainly dependent on the MyD88 signaling pathway in BMDM cells following GAS or *E. coli* infection.

Next, RAW cells were blocked with anti-TNFR1 monoclonal antibody prior to GAS infection. Data showed that A20 expression was slightly decreased when the dose of anti-TNFR1 antibody was 10 μg/ml or less (5 μg/ml), and was even higher following blockage with a fold dose (20 μg/ml) than the negative control (*P* < 0.001), although significantly reduced expression was observed (Figure 6B), indicating that GAS-induced A20 expression is partially dependent on TNFR1.

### The M protein of GAS plays an essential role in the induction of the A20 gene

**Figure 7:**
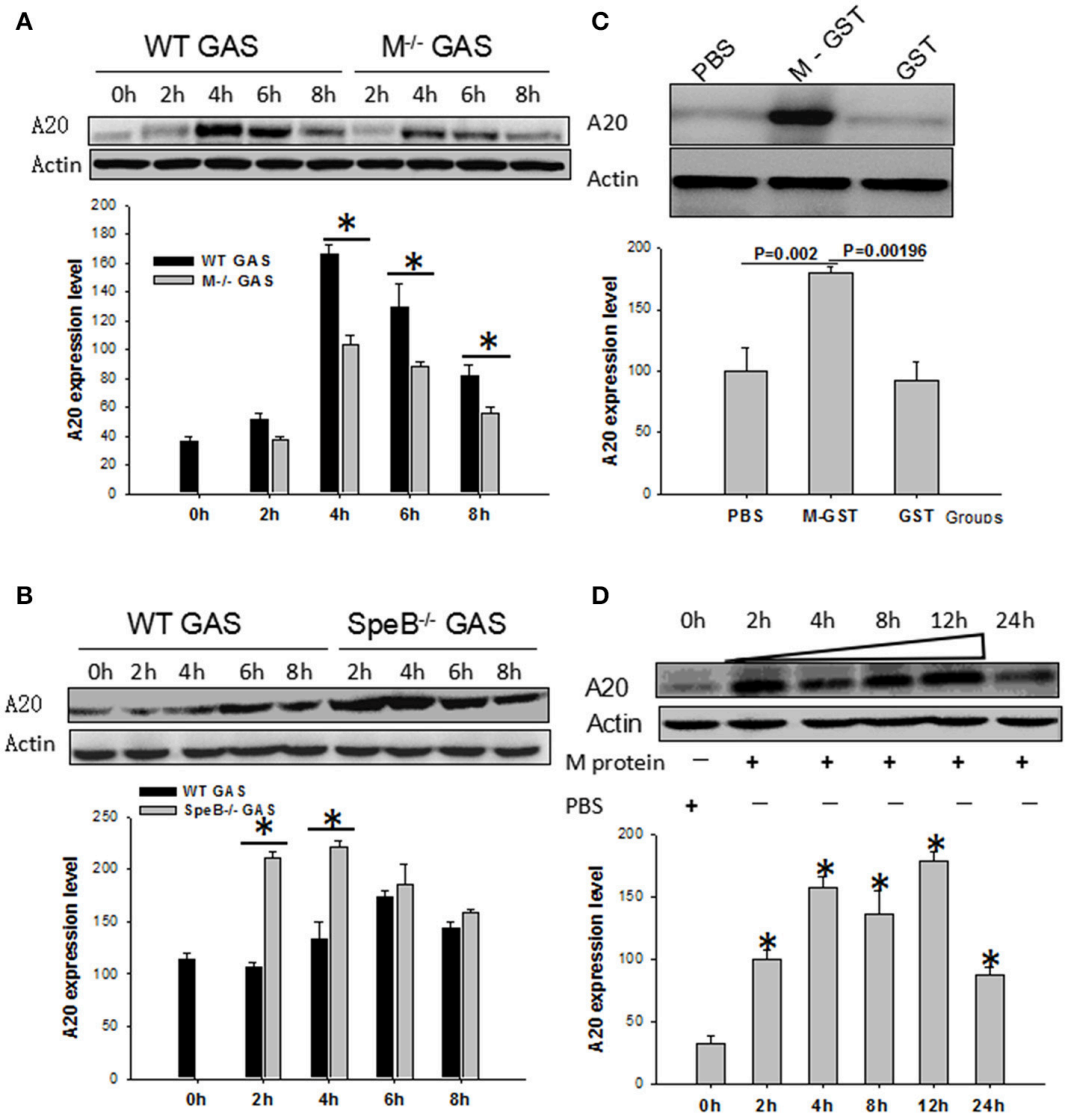
WT RAW cells induced by gene mutant strain M^−/−^ or SpeB^−/−^ GAS, or WT GAS, or purified M protein. RAW cells were stimulated by WT or M^−/−^ **(A)** or SpeB^−/−^GAS **(B)** at an MOI of 10. Six hours later, the treated cells were collected and A20 expression was determined by Western blotting. ^*^P < 0.05 vs. the M^−/−^ GAS group. RAW cells were stimulated by recombinant M-GST protein, GST-tag alone, and PBS as a control **(C)**, or recombinant purified M protein and PBS as a control **(D)**. Six hours later, the treated cells were collected and A20 expression was determined by Western blotting. Each experiment was repeated three times, and similar results were obtained on each occasion.

Because, the M protein of GAS plays an important role in pathogenic mechanisms, we used qPCR and Western blotting to evaluate its role on the induction of the A20 gene in BMDM cells. Interestingly, we found that the expression of A20 induced by M^−/−^ GAS was much lower than with WT GAS (*P* < 0.05; Figure 7A). Streptococcal erythrogenic toxin B (SpeB), a cysteine protease, is capable of degrading M protein. Consequently, we investigated BMDM cells that had been infected with SpeB^−/−^ GAS; Interestingly, A20 expression was vastly improved compared with WT GAS (Figure 7B), suggesting that the M protein might be related to GAS-induced A20 expression. To validate this supposition, we used M-GST protein to stimulate BMDM cells for 6 h to further test its function in terms of A20 gene induction, and found that M-GST induced higher A20 expression than the GST tag (*P* < 0.00196)or PBS control (*P* < 0.002; Figure 7C). Subsequently, we removed the GST tag from the M protein, and then used it to stimulate BMDM cells, and measure A20 expression over different time points. Results showed that high levels of A20 expression appeared from 2 to 12 h (Figure 7D), demonstrating that it is the M protein of GAS that plays an essential role in the expression of A20 by macrophages.

## Discussion

GAS is an important clinical pathogen that can cause a wide spectrum of diseases in humans, including pharyngitis, necrotizing fasciitis, erysipelas, toxic shock syndrome, sepsis, and even mortality. Furthermore, there is no effective prophylactic vaccine against GAS. Hence, it is very important to explore mechanisms underlying pathogenesis and resistance to immunity in order to identify novel therapeutic targets. The pathogenic effect of GAS is related to its strong toxicity and resistance to immunity (Horstmann et al., 1988), which is partially attributable to the GAS-mediated up-regulation of negative regulators in host cells. Host innate immune mechanisms play an essential role in controlling GAS growth and limiting the further spread of a pathogen beyond the site of infection (Goldmann et al., 2005). Resident macrophages have been shown to be critical in controlling infection in a mouse model (Goldmann et al., 2004). Generally, macrophages are activated by phagocytosis and express proinflammatory mediators such as TNF-α, IL-1β, and IL-6 to recruit phagocytic cells, such as neutrophils, more macrophages, and dentritic cells, which, in turn, induce inflammation at local sites (Park et al., 2008; Netea et al., 2009; Rojas et al., 2010). Upon activation of macrophages by a pathogen, multiple signaling cascades are triggered, leading to activation of nuclear factor-κB (NF-κB) which induces the expression of genes associated with proinflammatory cytokines and chemokines (Topley et al., 1996). Considering that NF-κB activation is central to many cellular processes and is closely-related to the expression of inflammatory cytokines, we analyzed NF-κB activation and the expression of proinflammatory mediators in macrophages infected with GAS. Surprisingly, results showed that compared with *E. coli*, GAS induced a lower expression level of inflammatory cytokines within 7 h of stimulation, and slight inflammation in the lung (Figure 2A). Accordingly, our previous results showed that the level of NF-κB activation induced by GAS was much lower than that by *Staphylococcus aureus*, but was similar to that induced by *E. coli* (Wu et al., 2016). It was not clear as to why the levels of inflammatory mediators induced by GAS were much lower than those induced by *E. coli*. However, we speculated that negative feedback proteins potentially played an essential role in the regulation of inflammatory mediators. To address this question, we investigated the expression of such negative factors, including SOCS-1 (Wu et al., 2015), SOCS-3, and A20. In particular, A20 is known to play a key role in the termination of NF-κB signaling, and inhibits activation of NF-κB (Boone et al., 2004; Wertz et al., 2004). TNF receptor associated factor (TRAF) 6, which mediates interaction with the NF-κB essential modulator (NEMO), is a target protein for A20 and can be degraded by the deubiquitination of A20, resulting in the inhibition of NF-κB signals (Hitotsumatsu et al., 2008). The expression of A20 can be induced by various stimulators of inflammation, including bacterial endotoxins, exotoxins, and proinflammatory cytokines (TNF-α and IL-1). In turn, A20 can inhibit the excessive release of various inflammatory factors and adhesion molecules (Wertz et al., 2004; Liu et al., 2005). Our present results showed that the level of A20 expression induced by GAS was much higher than that induced by *E. coli* in RAW264.7 cells or BMDM, accordingly, the expression level of the target protein TRAF6 was lower than that induced by *E. coli*. Thus, the activation of NF-κB was obviously inhibited from 2 to 6 h after GAS stimulation, which led to a reduction in the expression of proinflammatory cytokines. To further demonstrate these points, we next performed A20 SiRNA analysis, which showed that the level of p-P65 was significantly increased (Figures 4B,C), importantly, both mRNA and protein levels of proinflammatory cytokines were also significantly increased after silencing of the A20 gene, as shown in Figure 5.

MyD88 is an important adaptor protein in inflammatory pathways and signaling pathways mediated by Toll-like receptors (TLRs). With the exception of TLR3, all TLRs are MyD88 dependent (Kawai and Akira, 2010). We infected MyD88^−/−^ BMDMs with GAS to further investigate the correlation between MyD88 and A20, and found that there was no A20 expression in MyD88 knock-out cells, while A20 was detected readily in WT BMDM cells. This suggests that A20 expression is MyD88 dependent in both GAS- or *E. coli*-infected BMDM cells. However, there was no significant difference between the two groups in WT BMDM cells, indicating that MyD88 is not the main reason for differential A20 expression between the two groups.

In a previous study, Dixit et al. reported that binding of TNF-α to its receptor TNFR1 is the main signaling pathway mediating A20 expression after cytokine synthesis (Dixit et al., 1990; Opipari et al., 1992). In the present study, we detected high expression of TNF-α in GAS-induced macrophages. However, the blockade of RAW cells with anti-TNFR1 antibody showed that the expression of A20 was only partially blocked in GAS-infected RAW cells, indicating that the expression of A20 was partially dependent on the TNFR1 pathway. Consequently, we analyzed some important surface proteins of GAS in order to evaluate whether they had an effect on A20 expression. M protein and streptococcal erythrogenic toxin B (SpeB) of GAS were included in this analysis, largely because M protein is the most important membrane protein and the key virulence factor of GAS (Lancefield, 1962). This protein can exhibit a strong proinflammatory effect and contributes to the way in which GAS can evade phagocytosis via macrophages (Ma et al., 2009; Metzgar and Zampolli, 2011). SpeB is a cysteine protease known to cleave M protein from GAS (Raeder et al., 1998; Chaussee et al., 2000; Wei et al., 2005), while M protein affects the maturation of SepB. Interestingly, we found that A20 expression induced by M^−/−^GAS was significantly decreased compared with that induced by WT GAS. Instead, SpeB^−^GAS induced A20 expression was much higher than that of WT GAS in BMDM cells (Figures 7A,B), suggesting that when the SpeB gene of GAS was knocked out, the stability of the M protein was enhanced, thus leading to a higher level of A20 expression than that induced by WT GAS. This theory was proven by using purified M protein to stimulate macrophages (Figures 7C,D).

**Figure 8:**
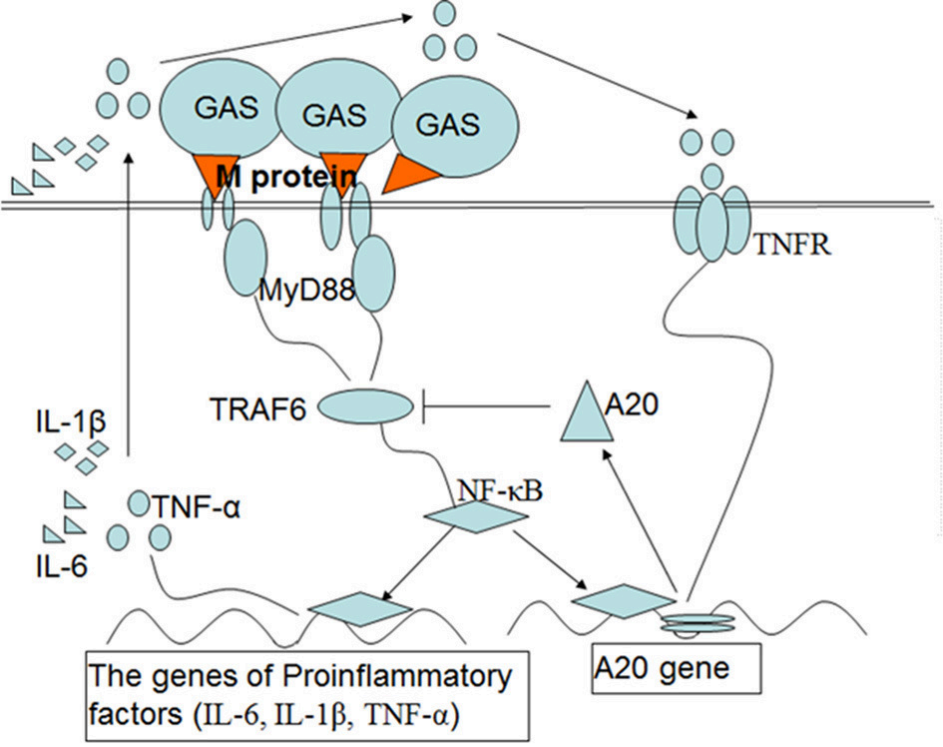
Schematic diagram of the signaling pathways that are postulated to upregulate the expression of A20 and then inhibit inflammatory mediators in macrophages induced by M protein following GAS infection. Upon induction of macrophages by M protein of GAS, NF-κB is activated through the MyD88 pathway, which induces expression of genes associated with the proinflammatory cytokines IL-1β, IL-6, and TNF-α. A20 expression depends on activation of NF-κB and partly on the interaction of TNF-αand TNFR. In turn, A20 targets TRAF-6 to inhibit inflammatory signal pathways, which leads to a reduction in the production of proinflammatory cytokines.

In summary, GAS induces a high level of A20 expression through its surface M protein which inhibits the inflammatory signal pathways, thus leading to a decrease in the production of proinflammatory cytokines (Figure 8). Thus, fewer inflammatory cells were recruited to the local infection site, which, in turn, might be conducive to GAS growth and further spread of the pathogen beyond the site of infection. In a previous paper, Liu et al. reported that the E3 ubiquitin ligase, TRIM29, inhibited interferon-regulatory factors and signaling via the transcription factor NF-κB of alveolar macrophages following infection with virus such as influenza virus (Xing et al., 2016). Collectively, our data and other studies implied that the function of macrophage could be regulated by several regulatory factors when infected by pathogens. This may have essential implications for our understanding of pathogenesis, as well as interactions between the host and the pathogen.

## Author contributions

LW and CM conceived and designed the experiments. CM, XG, and SW performed the experiments. LZ, JW, ZZ, ZY, and XS analyzed the data. WL, XW, and HF contributed reagents, materials, analysis tools. CM and XG wrote the manuscript. All authors read and approved the final version of the manuscript.

### Conflict of interest statement

The authors declare that the research was conducted in the absence of any commercial or financial relationships that could be construed as a potential conflict of interest.
